# Differences in both expression and protein activity contribute to the distinct functions of AINTEGUMENTA compared with AINTEGUMENTA-LIKE 5 and AINTEGUMENTA-LIKE 7

**DOI:** 10.1007/s11103-023-01374-0

**Published:** 2023-08-22

**Authors:** Beth A. Krizek, Caitlin Boling Iorio, Kevin Higgins, Han Han

**Affiliations:** 1 Department of Biological Sciences, University of South Carolina, Columbia, SC 29208 USA; 2 Metagenetics LLC, Columbia, SC 29205 USA; 3 Co-Innovation Center for Sustainable Forestry in Southern China, College of Biology and the Environment, Nanjing Forestry University, Nanjing, 210037 China

**Keywords:** Arabidopsis, Flower development, AINTEGUMENTA, AIL5/PLT5, AIL7/PLT7

## Abstract

Three members of the Arabidopsis AINTEGUMENTA-LIKE/PLETHORA (AIL/PLT) transcription factor family, AIL5/PLT5, AIL6/PLT3, and AIL7/PLT7, exhibit partially overlapping roles with AINTEGUMENTA (ANT) during flower development. Loss of *ANT* function alone results in smaller floral organs and female sterility indicating that some ANT functions cannot be provided by these related transcription factors. Previously, we showed that expression of *AIL6* at the same levels and spatial pattern as *ANT* could largely rescue the defects of *ant* mutants. This suggested that the functional differences between *ANT* and *AIL6* were primarily a consequence of expression differences. Here, we investigated the functional differences between *ANT* and both *AIL5* and *AIL7* by expressing these two *AIL*s under the control of the *ANT* promoter. We found that only *ANT:gAIL5* lines with much higher amounts of *AIL5* mRNA as compared with *ANT* could compensate for loss of *ANT* function. *ANT:gAIL7* lines with *AIL7* mRNA levels similar to those of *ANT* were able to rescue some but not all aspects of the *ant* mutant phenotype. Thus, expression differences alone cannot explain the functional differences between ANT and these two related proteins. Studies in yeast show that AIL5 and AIL7 have lower transcriptional activation activities as compared with ANT and AIL6 when bound to the consensus ANT DNA binding site. Our results suggest that differences in both expression and protein activity contribute to the functional specificity of ANT compared with AIL5 and AIL7.

**Supplementary Information:**

The online version contains supplementary material available at 10.1007/s11103-023-01374-0.

## Key message

The distinct contributions of the related Arabidopsis transcription factors ANT, AIL5, and AIL7 in flower development result from differences in both expression and protein activity.

**Supplementary Information:**

The online version contains supplementary material available at 10.1007/s11103-023-01374-0.

## Introduction

Flower development in *Arabidopsis thaliana* is an important model system for understanding how cell division and differentiation are controlled to create floral organs of defined morphology and size. Floral meristems arise from the periphery of the dome-shaped inflorescence meristem during reproductive development. These floral meristems give rise to floral organ primordia in four concentric whorls. Floral organ primordia adopt different fates in each whorl based on the activity of distinct combinations of floral organ identity genes as described by the ABCE model [reviewed in (Thomson et al. 2016)]. Class A and E gene activities in whorl one specify sepal identity, class A, B and E gene activities in whorl two specify petal identity, class B, C, and E gene activities in whorl three confers stamen identity, and class C and E activities in whorl four specify carpel identify. Most of the class A, B, C, and E floral organ identity genes encode MADS domain transcription factors while the class A gene, *APETALA2*, encodes a founding member of the APETALA2/ETHYLENE RESPONSE FACTOR (AP2/ERF) transcription factor family (Yanofsky et al. 1990; Jack et al. 1992; Mandel et al. 1992; Goto and Meyerowitz 1994; Okamuro et al. 1997).

Four members of the AINTEGUMENTA-LIKE/PLETHORA (AIL/PLT) subfamily of AP2/ERF transcription factors: AINTEGUMENTA (ANT), AIL5, AIL6, and AIL7 also play important roles in floral organogenesis. *ANT* and *AIL6* have partially overlapping roles in preventing premature differentiation of the floral meristem, regulating floral organ initiation and positioning, specifying organ identity, and promoting organ growth (Krizek 2009; Krizek and Eaddy 2012; Krizek et al. 2021). *AIL6* cannot substitute for *ANT* in all of these roles as mutations in *ANT* result in smaller floral organs, reductions in floral organ numbers, and female sterility (Elliott et al. 1996; Klucher et al. 1996). Loss of *AIL6* alone has no phenotypic effect (Krizek 2009) indicating unequal genetic redundancy between *ANT* and *AIL6* (Briggs et al. 2006). Importantly, we have found that AIL6 binds a subset of the genomic sites bound by ANT suggesting that AIL6 can regulate many but not all of the downstream regulatory targets of ANT (Krizek et al. 2021). Differences in the functions of ANT and AIL6 appear to result primarily from differences in gene expression. *ANT* is expressed at much higher levels than *AIL6* and in a broader domain that persists longer during floral organ development (Elliott et al. 1996; Nole-Wilson et al. 2005). Expression of *AIL6* under the control of the *ANT* promoter largely complements an *ant* mutant indicating that AIL6 can provide ANT function when expressed at the same levels and places as ANT (Han and Krizek 2016).

AIL5 and AIL7 make smaller contributions to floral organ development as compared with ANT and AIL6. Like *AIL6*, mutations in *AIL5* or *AIL7* have no phenotypic effect on their own but these mutations enhance *ant* single mutants (Krizek 2015). Our previous genetic results suggest that *AIL5* has overlapping roles with *ANT* in sepal positioning and petal initiation and growth, while *AIL7* has overlapping roles with *ANT* in sepal positioning, petal initiation, and carpel fusion (Krizek 2015). The different functions of ANT, AIL5, and AIL7 could result from differences in expression and/or from differences in protein activity. To investigate the molecular basis for their different functions, we examined the ability of genomic copies of *AIL5* and *AIL7* to provide *ANT* function when expressed under the control of the *ANT* promoter. As *AIL5* is expressed in a similar pattern as *ANT* in flowers up to stage 6 (Elliott et al. 1996; Nole-Wilson et al. 2005), this approach would be expected to primarily result in *AIL5* overexpression. In the case of *AIL7*, expression conferred by the *ANT* promoter will result in both ectopic expression and overexpression. *AIL7* is expressed primarily in the centermost floral meristem cells of stage 2–5 flowers with later expression in the microsporangia of anthers and placenta of carpels, and thus shows a far more restricted pattern of expression as compared with *ANT* (Elliott et al. 1996; Nole-Wilson et al. 2005).

Characterization of *ANT:gAIL5 ant* and *ANT:gAIL7 ant* transgenic plants showed that increased expression of *AIL5* and *AIL7* can compensate for some aspects of the *ant* loss of function phenotype. For *AIL5*, recovery of all aspects of the *ant* phenotype required higher *AIL5* mRNA levels than corresponding *ANT* levels in wild-type plants. For *AIL7*, a line expressing *AIL7* at approximately the levels of *ANT* in wild-type plants also rescued some but not all aspects of the *ant* mutant phenotype. In this case, higher *AIL7* mRNA levels resulted in dramatic changes in flower development including the production of bracts and mosaic floral organs, reduced numbers of floral organs, and fusion of stamens to central carpelloid structures. We found some of these same phenotypes in an ethanol inducible *AIL7* overexpression line. Our results suggest that differences in expression levels and spatial pattern do not fully explain the distinct functions of *AIL5* and *AIL7* compared with *ANT*. Thus, differences in protein activities likely also contribute to their distinct functions. We found that AIL5 and AIL7 exhibit reduced transcriptional activation activity in yeast cells as compared with ANT and AIL6 when the reporter gene was under the control of ANT DNA binding sites. Thus, differences in transcriptional activation and/or differences in DNA binding affinity and/or specificity may contribute to the functional differences between these proteins.

## Materials and methods

### Plant materials and growth conditions

The *ant-4* allele was genotyped as described previously (Krizek 2009). Plants were grown on a soil mixture of either Metro-Mix 360:perlite:vermiculite (5:1:1), Fafard 4P:perlite:vermiculite (8:1:1), or SunGro Sunshine Mix #1:vermiculite:perlite: (8:1.5:0.5) in 16 h days (100–150 μmol·m^− 2^·s^− 1^) at 22 °C.

### Plasmid construction and plant transformation

A genomic copy of *AIL5* containing the entire *AIL5* gene and 500 base pairs of 3’ sequence was cloned into BJ97. A 6.2 kb *ANT* upstream promoter sequence was cloned into the Kpn site of the AIL5-3’/BJ97 construct. This promoter sequence is sufficient to complement *ant-4* when fused to the coding sequence of *ANT* (Krizek 2009). *ANT:gAIL5-3’* was subcloned into the NotI site of both pART27 and pMLBart and transformed into *Agrobacterium tumefaciens* strain ASE by electroporation. A genomic copy of *AIL7* containing the entire *AIL7* gene and 503 base pairs of 3’ sequence was cloned into BJ36. The 6.2 kb *ANT* promoter was cloned into the Kpn site of this AIL7-3’/BJ36 construct. *ANT:gAIL7-3’* was subcloned into the NotI site of pMLBart and transformed into *Agrobacterium tumefaciens* strain ASE by electroporation. *ant-4* plants were transformed with these *Agrobacterium* strains by vacuum infiltration (Bechtold et al. 1993). Transformants were selected for either kanamycin resistance (pART27) or basta resistance (pMLBart). For the ethanol inducible *AIL7* construct, a genomic copy of *AIL7* containing 41 base pairs of 5’ sequence, the entire *AIL7* gene and 503 base pairs of 3’ region was cloned into the PstI and BamHI sites of BJ36_AlcA (Maizel and Weigel 2004). *AlcA:gAIL7* was subcloned into the NotI site of pAM54 which contains the *LEAFY* (*LFY*) promoter driving the *AlcR* gene in pMLBart (Maizel and Weigel 2004). *LFY:AlcR*/*AlcA:gAIL7*/pMLBart was transformed into *Agrobacterium* strain ASE by electroporation. Transformants were selected for basta resistance.

### Petal size measurement

Petal measurements were performed as described previously (Trost et al. 2014). Petals from approximately stage 13 flowers were removed with forceps and placed on Sellotape. After petals were collected, the tape was adhered to a piece of black plexiglass and scanned at a resolution of 3600 dpi in 8-bit greyscale. Petal area, length and width were determined using Image J software. Measurements were performed on at least 20 petals from flowers at positions 1–10 on inflorescences from at least 4 different plants.

### Scanning electron microscopy

Tissue for SEM was fixed, dehydrated, dissected and coated as previously described (Krizek 1999). SEM analyses were performed on a Tescan Vega-3 SBU Variable Pressure SEM.

### RNA extraction and RT-qPCR

RNA was extracted from inflorescences using TRIzol (Life Technologies) and treated with DNase while on an E.Z.N.A. Plant RNA spin column (Omega Bio-Tek). First-strand cDNA synthesis was performed using either qScript cDNA Supermix kit (Quanta BioSciences) or iScript cDNA Synthesis Kit (Bio-Rad). Real-time PCR reactions were performed on a Bio-Rad CFX Connect using either PerfeCTa SYBR Green FastMix for iQ (Quanta BioSciences) or iQ SYBR Green Supermix (Bio-Rad). Data analyses were carried out using the 2^− ΔΔCt^ method (Livak and Schmittgen 2001). Normalization was performed using AT5G15710 as a reference gene (Czechowski et al. 2005). Three biological replicates were used in each experiment.

### In situ hybridization

Inflorescences were fixed, embedded, sectioned, hybridized and washed as described in (Wu and Wagner 2012). Probes were made using the Riboprobe in vitro transcription T7 system (Promega) and DIG-UTP (Roche) on templates derived from PCR products. For the *AIL5* template, *AIL5* was PCR amplified with AIL5-46 (5’-GATGGGTCACCGGGAGTT-3’) and AIL5-T7 (5’-CATAATACGACTCACTATAGGGTCCACCATACCCTTCGTTACC-3’). For the *AIL7* template, *AIL7* was PCR amplified with AIL7-FW (5’-CCAGATTTCAAGACGATAAACTC-3’) and AIL7-T7 (5’-CATAATACGACTCACTATAGGGTCTGGTGGTAATAGAGAACTGA-3’).

### Ethanol induction

For floral organ counts and pictures, 14 day old *LFY:AlcR/AlcA:gAIL7* plants were treated with mock (H_2_O) or ethanol vapor by placing 2 ml of water or 100% ethanol in one microfuge tube in each of half of the pots in the tray. For expression experiments (RT-qPCR and in situ hybridization), 26–28 day old *LFY:AlcR/AlcA:gAIL7* plants were treated with mock (H_2_O) or ethanol vapor by placing 2 ml of water or 100% ethanol in one microfuge tube in every pot in the tray. Trays was covered with plastic dome lids for eight hours.

### Yeast constructs, transformation, and β-galactosidase assays

ANT, AIL5, AIL6 and AIL7 were first tested for transcription activation activity when fused to the GAL4 DNA binding domain (GBD) in pGBT9. Clones corresponding to the coding regions of AIL5, AIL6, and AIL7 were cloned into the SmaI site of pGBT9. These GBD-AIL constructs as well as the previously described GBD-ANT construct were transformed into the yeast strain HF7c in which the *lacZ* reporter gene is under the control of three GAL4 binding sites and the TATA portion of the CYC1 promoter (Krizek and Sulli 2006). Transformants were selected on plates containing synthetic medium lacking tryptophan and tested for their ability to activate the *lacZ* gene. ANT, AIL5, AIL6, and AIL7 were also tested for their ability to activate the *lacZ* gene when it was under the control of three copies of the ANT consensus binding site (yeast strain BK1) (Krizek 2003). In this case, the coding regions of *AIL5*, *AIL6*, and *AIL7* (lacking the stop codons) were cloned into pGAD424 in which the GAL4 activation domain was removed. These constructs retain the SV40 T-antigen nuclear localization signal and stop codons were provided by the plasmid. The *AIL5* coding region was cloned into the KpnI and BamHI sites of pGAD424. The *AIL6* coding region was cloned into the KpnI and SmaI sites of pGAD424. The *AIL7* coding region was cloned into the KpnI and SalI sites of pGAD424. The ANT/pGAD424 construct was described previously (Krizek 2003). These plasmids were transformed into the BK1 yeast strain and transformants were selected on plates containing synthetic medium lacking leucine. β-galactosidase assays were performed as described previously (Krizek 2003).

### PADDLE (predictor of activation domains using deep learning in Eukaryotes)

PADDLE was downloaded from github (https://github.com/asanborn/PADDLE) (Sanborn et al. 2021). Details about running PADDLE and the PADDLE output are available at http://bitbucket.org/krizeklab.

## Results

### Increased expression of ***AIL5*** can complement ***ant***

**Fig. 1:**
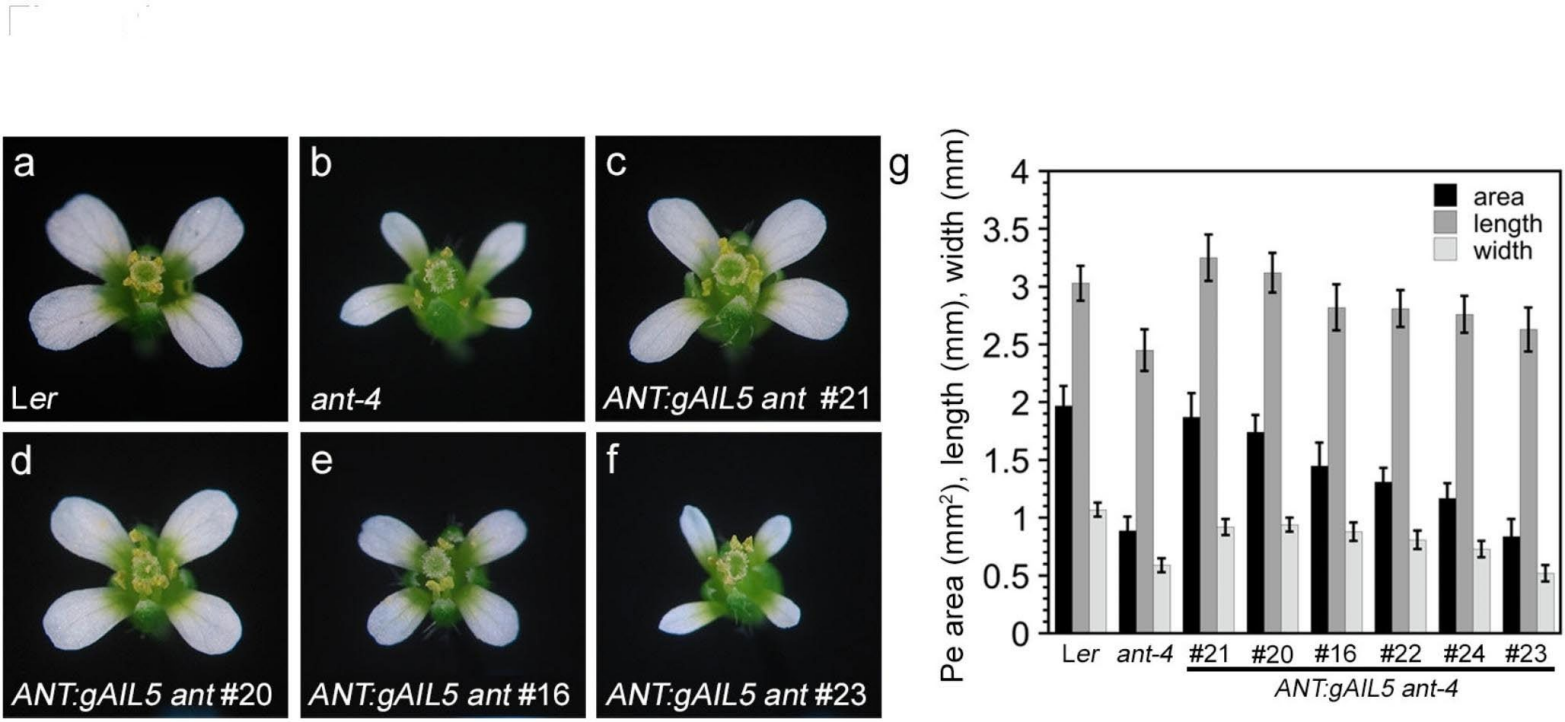
*ANT:gAIL5 ant* lines show varying degrees of complementation of *ant-4* petal growth (**a**) L*er* flower, (**b**) *ant-4* flower, (**c**) *ANT:gAIL5 ant-4* line 21 flower, (**d**) *ANT:gAIL5 ant-4* line 20 flower, (**e**) *ANT:gAIL5 ant-4* line 16 flower, (**f**) *ANT:gAIL5 ant-4* line 23 flower, (**g**) Petal (Pe) area, length, and width for L*er*, *ant-4*, and *ANT:gAIL5 ant-4* flowers. All flower pictures were taken at the same magnification

We investigated whether increased expression of *AIL5*, as conferred by the *ANT* promoter, could complement the *ant* mutant phenotype. We generated six *ANT:gAIL5 ant-4* transgenic lines in which a genomic copy of *AIL5* was expressed under the control of the *ANT* promoter. *ant-4* and *ANT:gAIL5 ant-4* flowers were compared with regard to four characteristics of the *ant* mutant phenotype: petal size, anther locule number, floral organ number, and seed production. All of these phenotypes likely reflect the role of ANT in promoting growth, either in floral meristems, floral organ primordia, or ovule primordia (Elliott et al. 1996; Klucher et al. 1996; Baker et al. 1997; Krizek 1999). *ant-4* flowers produce smaller petals than wild type, stamen anthers that are composed of only two locules instead of four, and fewer floral organs (primarily due to reduced numbers of stamens) (Fig. 1a, b, g; Supplementary Fig. S1a, b; Table 1). In addition, *ant-4* flowers are female sterile because of a defect in embryo sac development and do not produce seeds. The defect in embryo sac development is thought to be a consequence of reduced growth of the integuments within the ovule (Baker et al. 1997).

**Table 1:** Floral organ counts in L*er* and *ANT:gAIL5 ant-4* lines

	L*er*	*ant-4*	line 21	line 20	line 16	line 22	line 24	line 23
**Whorl 1**								
Se	4.00	3.97	3.99	4.00	4.00	3.99	3.82	4.00
filament		0.01						
**total**	**4.00**	**3.98**	**3.99**	**4.00**	**4.00**	**3.99**	**3.82**	**4.00**
**Whorl 2**								
Pe	4.00	3.83	3.92	4.00	4.00	3.99	3.48	3.89
Se/Pe		0.01						
filament			0.01				0.04	0.02
**total**	**4.00**	**3.84**	**3.93**	**4.00**	**4.00**	**3.99**	**3.52**	**3.91**
**Whorl 3**								
St	5.62	4.63	5.57	5.06	4.75	4.60	4.81	4.51
St-like	0.02	0.05	0.03	0.01	0.02		0.04	0.03
filament	0.01	0.01		0.01		0.03	0.03	0.02
**total**	**5.65**	**4.69**	**5.60**	**5.08**	**4.77**	**4.63**	**4.88**	**4.56**
**Whorl 4**								
Ca	2.00	2.00	2.00	2.00	2.00	2.00	2.00	2.00
**total**	**2.00**	**2.00**	**2.00**	**2.00**	**2.00**	**2.00**	**2.00**	**2.00**
**Total all whorls**	**15.65**	**14.51**	**15.52**	**15.08**	**14.77**	**14.61**	**14.22**	**14.47**

The six *ANT:gAIL5* lines varied in their ability to complement different aspects of the *ant-4* phenotype. Two lines exhibited complete (line 21) or nearly complete (line 20) rescue of all *ant* phenotypic defects while three lines showed partial rescue (lines 16, 22, 24) and one line showed no rescue (line 23). *ANT:gAIL5 ant* line 21 plants produced petals similar in area to wild type petals, stamens with four locules, similar numbers of floral organs as wild type, and were fertile (Fig. 1c, g; Supplementary Fig. S1c; Tables 1 and 2). *ANT:gAIL5 ant* line 20 rescued anther locule number and seed set with slightly less rescue of petal size and floral organ number than line 21 (Fig. 1d, g; Tables 1 and 2). *ANT:gAIL5 ant* lines 16, 22, and 24 rescued stamen locule number and partially rescued petal area (Fig. 1e, g; Tables 1 and 2). These lines did not rescue floral organ number or seed production (Tables 1 and 2). *ANT:gAIL5 ant* line 23 flowers resembled *ant-4* (Fig. 1f; Supplementary Fig. S1d; Tables 1 and 2).

**Table 2:** Summary of *ant-4* complementation by *ANT:gAIL5, ANT:gAIL7*, and *ANT:gAIL6*

	Pe area (% of L*er*)	locule #	# floral organs (% of L*er*)	seeds	rel. *AIL* exp.
L*er*	100	4	100	yes	
*ant-4*	45.2	2	92.7	no	0.75 ± 0.07
*ANT:gAIL5 ant-4* #21	94.9	4	99.2	yes	17.56 ± 1.7
*ANT:gAIL5 ant-4* #20	88.3	4	96.4	yes	8.70 ± 0.85
*ANT:gAIL5 ant-4* #16	73.6	4	94.4	no	3.63 ± 0.20
*ANT:gAIL5 ant-4* #22*	66.5	4	93.3	no	3.15 ± 0.01
*ANT:gAIL5 ant-4* #24	59.4	4	90.9	no	3.24 ± 0.40
*ANT:gAIL5 ant-4* #23	42.6	2	92.5	no	4.15 ± 0.73
L*er*	100	4	100	yes	
*ant-4*	50.5	2	94.2	no	1.02 ± 0.05
*ANT:gAIL7 ant-4* #10	104.1	4	84.3	no	35.40 ± 5.70
*ANT:gAIL7 ant-4 #*3*	77.3	4	93.6	yes	8.87 ± 0.94
*ANT:gAIL7 ant-4 #*12	75.8	4	94.9	yes	7.11 ± 1.26
*ANT:gAIL7 ant-4 #*9	67.0	4	93.1	no	5.69 ± 0.32
*ANT:gAIL7 ant-4 #*2	48.5	2	92.2	no	1.15 ± 0.09
L*er*	100	4	100	yes	
*ant-4*	46.3	2	92.3	no	1.43 ± 0.03
*ANT:gAIL6 ant-4* C-69*	88.1	4	97.0	yes	9.00 ± 0.44

* Lines that express the respective *AIL* gene (*AIL5*, *AIL7*, or *AIL6*) at mRNA levels most similar to *ANT* mRNA levels in wild-type plants

### The degree of rescue of ***ant*** by ***ANT:gAIL5*** is correlated with ***AIL5*** mRNA levels

**Fig. 2:**
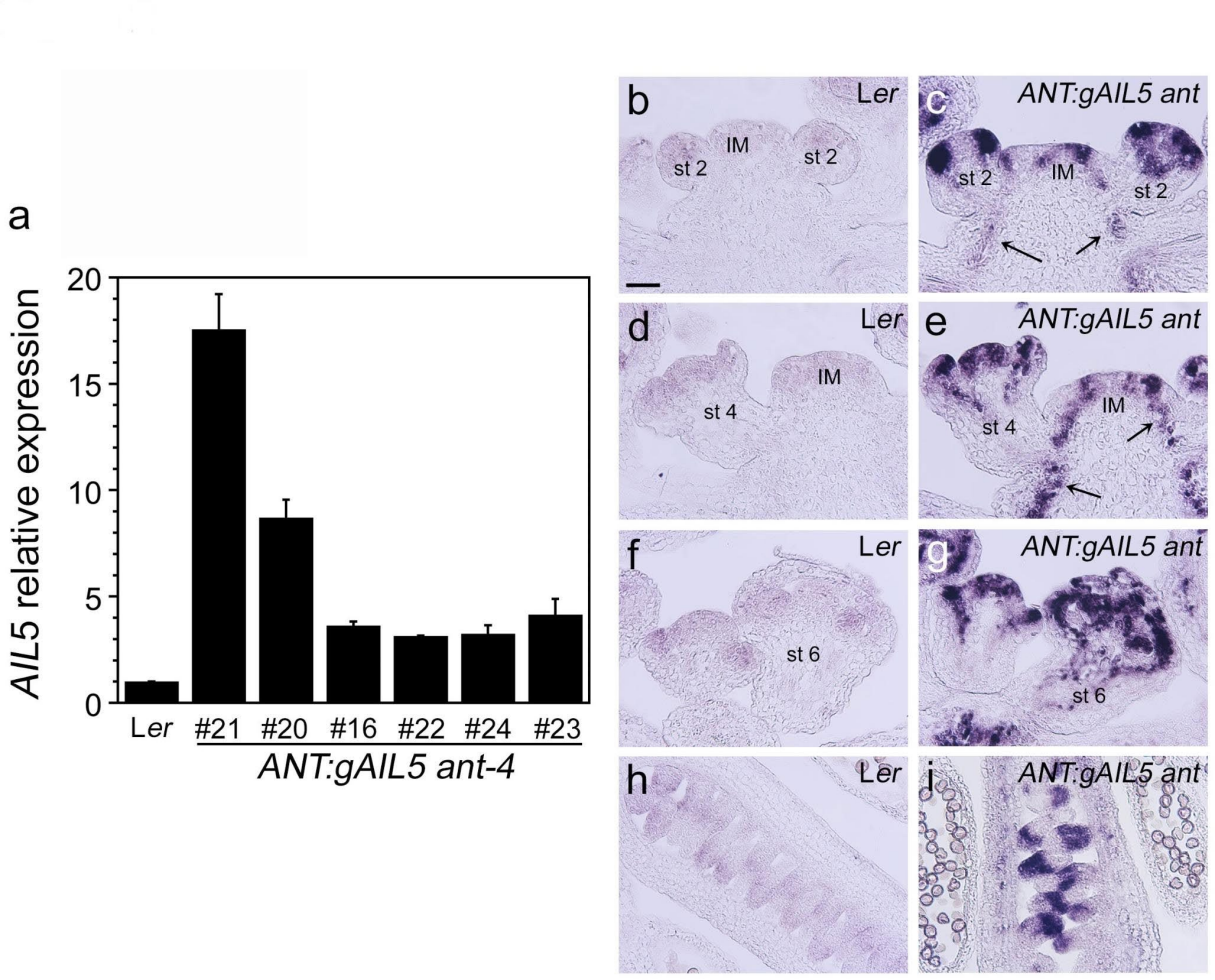
*AIL5* expression is increased in *ANT:gAIL5 ant* lines. **a**. *AIL5* mRNA expression in L*er* and *ANT:gAIL5 ant-4* lines determined by RT-qPCR, **b**-**i**. In situ hybridization of *AIL5* mRNA in L*er* (**b**, **d**, **f**, **h**) and *ANT:gAIL5 ant* line 21 (**c**, **e**, **g**, **i**) inflorescences. Arrows in c and e point to *AIL5* expression in the procambium. Abbreviations: IM, inflorescence meristem; st 2, stage 2 flower; st 4, stage 4 flower; st 6, stage 6 flower. Size bar corresponds to 50 μm

To determine whether phenotypic differences among *ANT:gAIL5 ant* lines might result from different levels of *AIL5* mRNA in the transgenic lines, we performed RT-qPCR on L*er* and *ANT:gAIL5 ant* inflorescences. *AIL5* mRNA levels were higher in lines 21 and 20 as compared with lines 16, 22, 24, and 23 (Fig. 2a; Table 2). *AIL5* mRNA levels were 17.6 fold higher in *ANT:gAIL5 ant* line 21 as compared with L*er* and 8.7 fold higher in *ANT:gAIL5 ant* line 20 as compared with L*er* (Table 2). *ANT:gAIL5 ant* lines 16, 22, 24, and 23 had similar levels of *AIL5* mRNA, about 3–4 fold higher than *AIL5* mRNA levels in L*er* (Table 2). These results indicate a correlation between *AIL5* mRNA expression levels and the degree of rescue, with higher levels conferring more rescue. An absolute RT-qPCR experiment showed that wild-type inflorescences have approximately 2.2-fold more copies of *ANT* mRNA as compared with *AIL5* mRNA. Thus *ANT:gAIL5 ant* line 22 corresponds to a line in which *AIL5* mRNA levels are most similar to ANT levels in wild-type plants. Only *ANT:gAIL5* transgenic lines 20 and 21, with much higher levels of *AIL5* (8–17 fold higher) provide similar biological functions as ANT.

We also examined the spatial expression pattern of *AIL5* mRNA in *ANT:gAIL5 ant* line 21 inflorescences by in situ hybridization. As expected, the spatial expression pattern of *AIL5* mRNA is similar in L*er* and *ANT:gAIL5 ant* inflorescences with a much stronger signal in the transgenic *ANT:gAIL5 ant* line (Fig. 2b-i). In addition, we see *AIL5* expression in the procambium of the inflorescence stem which is not seen in L*er* (Fig. 2b-e). This is consistent with the *ANT* promoter being active in this tissue (Elliott et al. 1996).

### Expression of ***AIL7*** under the control of the ***ANT*** promoter can partially complement ***ant***

**Fig. 3:**
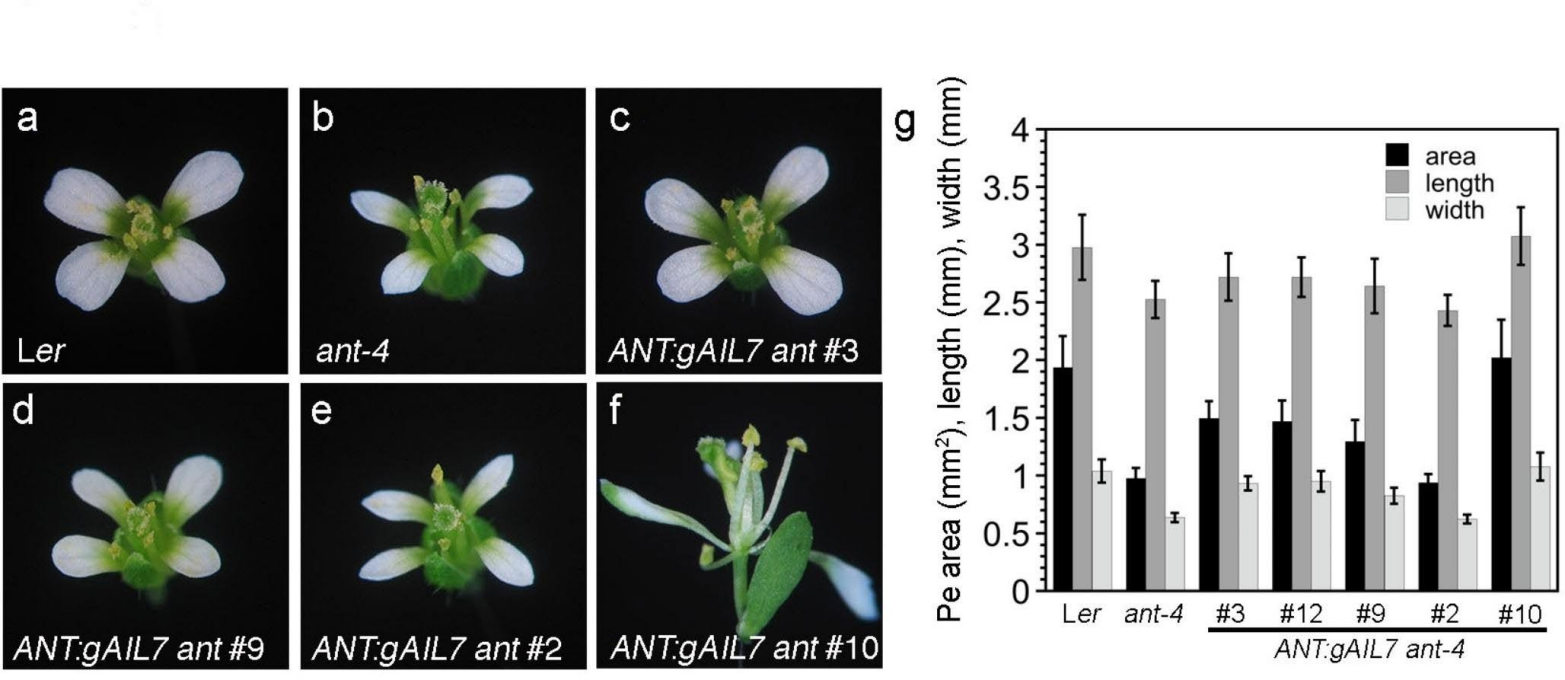
*ANT:gAIL7 ant* lines show varying degrees of complementation of *ant-4* petal growth (**a**) L*er* flower, (**b**) *ant-4* flower, (**c**) *ANT:gAIL7 ant-4* line 3 flower, (**d**) *ANT:gAIL7 ant-4* line 9 flower, (**e**) *ANT:gAIL7 ant-4* line 2 flower, (**f**) *ANT:gAIL7 ant-4* line 10 flower, (**g**) Petal (Pe) area, length, and width for L*er*, *ant-4*, and *ANT:gAIL7 ant-4* flowers. All flower pictures were taken at the same magnification

To investigate whether *AIL7* can complement *ant-4* when expressed under the control of the *ANT* promoter, we generated five *ANT:gAIL7 ant* transgenic lines. The *ANT:gAIL7* lines varied in their ability to complement the petal area, locule number, and fertility defects of *ant-4*. None of the *ANT:gAIL7* lines rescued the floral organ number defect of *ant-4* (Table 3). *ANT:gAIL7 ant* lines 3 and 12 rescued the anther locule number and fertility defects, but only partially rescued petal size (Fig. 3a-c, g; Supplementary Fig. S1e; Table 2). *ANT:gAIL7 ant* line 9 rescued anther locule number, partially rescued petal size but did not produce seeds (Fig. 3d, g; Table 2). The flowers of *ANT:gAIL7 ant* line 2 resembled *ant-4* (Fig. 3e, g; Supplementary Fig. S1f; Table 2).

**Table 3:** Floral organ counts in L*er* and *ANT:gAIL7 ant-4* lines 3, 12, 9, and 2

	L*er*	*ant-4*	line 3	line 12	line 9	line 2
**Whorl 1**						
Se	4.00	3.98	3.98	4.00	4.01	4.02
**total**	**4.00**	**3.98**	**3.98**	**4.00**	**4.01**	**4.02**
**Whorl 2**						
Pe	4.00	3.92	3.98	3.96	4.00	3.95
Pe/Se or Se/Pe						0.01
filament				0.01		
**total**	**4.00**	**3.92**	**3.98**	**3.97**	**4.00**	**3.96**
**Whorl 3**						
St	5.60	4.79	4.62	4.83	4.51	4.40
St-like		0.01	0.02	0.01	0.01	
filament						0.01
**total**	**5.60**	**4.80**	**4.64**	**4.84**	**4.52**	**4.41**
**Whorl 4**						
Ca	2.00	2.00	2.00	2.00	2.00	2.00
**total**	**2.00**	**2.00**	**2.00**	**2.00**	**2.00**	**2.00**
**Total all whorls**	**15.60**	**14.70**	**14.60**	**14.81**	**14.53**	**14.39**

*ANT:gAIL7 ant* line 10 flowers rescued anther locule number but had a number of additional phenotypes, some of which affected fertility, petal development and floral organ number. These included subtending bracts, mosaic floral organs, reduced numbers of florals organs, altered positioning of floral organs, carpelloid or stamenoid/carpeloid organs in the fourth whorl, and fusion between stamens and carpelloid organs (Fig. 3f; Table 4). Because of the absence of normal carpels in the fourth whorl of *ANT:gAIL7 ant* line 10 flowers, we could not obtain seeds when the transgene was homozygous (Table 2). Because the *ANT:gAIL7 ant* line 10 petals are reduced in number and often sepaloid or fused together, we performed petal area measurements on *ANT:gAIL7 ant* line 10 plants heterozygous for the transgene (Supplementary Fig. S2; Fig. 3f; Table 3). The petals of these hemizygous plants were similar in size to wild type (Fig. 3g; Table 2). Some of the additional floral phenotypes present in *ANT:gAIL7 ant* line10 flowers were similar to those previously observed in transgenic lines of *ANT:gAIL6 ant* that express *AIL6* at high levels (Han and Krizek 2016).

**Table 4:** Floral organ counts in L*er* and *ANT:gAIL7 ant-4* line 10

	L*er*	line 10 homo	line 10 hemi
**Outer Whorls**			
Se	4.00	0.32	2.95
Pe/Se		0.39	0.92
Se/Pe		0.59	0.27
Pe	4.00	1.18	2.12
filament		1.99	0.23
St; St-like		0.24	
St/Pe; Pe/St		0.10	
St-Se/Pe		0.01	
leaf-like		0.03	
**Inner Whorls**			
St	5.60	3.43	4.62
St-like		0.06	0.09
Ca	2.00	0.21	1.82
Ca-like		0.36	
St/Ca; Ca/St		0.35	0.02
filament		0.03	
**total all whorls**	**15.60**	**9.29**	**13.15**

### *AIL7* mRNA levels correlate with the amount of petal size rescue in ***ANT:gAIL7 ant*** lines

**Fig. 4:**
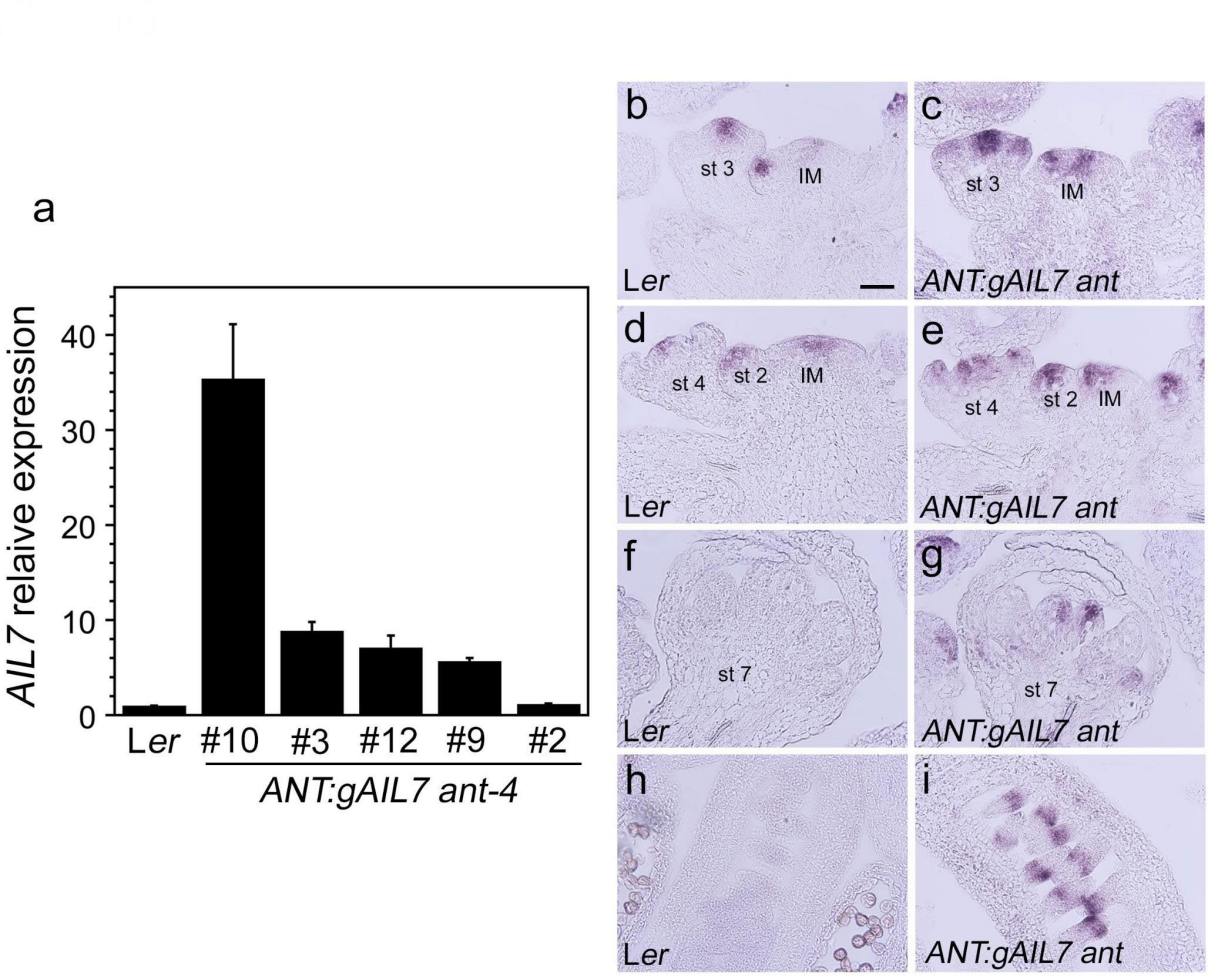
*AIL7* expression is increased in *ANT:gAIL7 ant* lines **a**. *AIL7* mRNA expression in L*er* and *ANT:gAIL7 ant-4* lines determined by RT-qPCR, **b-i**. In situ hybridization of *AIL7* mRNA in L*er* (**b, d, f, h**) and *ANT:gAIL7 ant* line 3 (**c, e, g, i**) inflorescences. Abbreviations: IM, inflorescence meristem; st 2, stage 2 flower; st 3, stage 3 flower; st 4, stage 4 flower; st 7, stage 7 flower. Size bar corresponds to 50 μm

To determine whether the phenotypic differences among the *ANT:gAIL7 ant* lines correlated with different *AIL7* mRNA expression levels, we performed RT-qPCR on inflorescences. For *ANT:gAIL7 ant* line 10, we collected inflorescences primarily from heterozygous plants with a few homozygous plants included. *AIL7* mRNA levels were highest in *ANT:gAIL7 ant* line 10 inflorescences, with 35.4-fold higher mRNA levels in this line as compared with wild type (Fig. 4a; Table 2). *AIL7* mRNA levels were 8.9, 7.1, 5.7, and 1.2-fold higher in *ANT:gAIL7 ant* lines 3, 12, 9, and 2 compared with L*er* (Fig. 4a; Table 2). These results show a direct correlation between *AIL7* mRNA expression levels and the degree of rescue, particularly with regard to petal size. Absolute RT-qPCR revealed that *ANT* mRNA levels are about 10-fold higher than *AIL7* mRNA in L*er* inflorescences. Thus, *ANT:gAIL7 ant* line 10 corresponds to an overexpression line while *ANT:gAIL7 ant* line 3 corresponds to a line in which *AIL7* mRNA levels are similar to *ANT* levels in wild type plants. When *AIL7* is expressed at levels similar to *ANT*, there was rescue of anther locule number and seed production and partial rescue of petal size. Only when *AIL7* is expressed at very high levels (35-fold greater than in *AIL7* in wild type), as in *ANT:gAIL7 ant* line 10, was there complete rescue of petal size. But this level of *AIL7* expression also had striking consequences on flower development with alterations in the positioning of floral organs and the production of mosaic and fused floral organs.

In situ hybridization shows that *AIL7* mRNA accumulates in a broader domain and at higher levels in *ANT:gAIL7 ant* line 3 flowers as compared with L*er* (Fig. 4b-i). *AIL7* mRNA is detected in sepal primordia of stage 3 and 4 flowers in *ANT:gAIL7 ant* line 3 but not L*er* (Fig. 4b-e). *AIL7* mRNA was also detected in petal and carpel primordia in *ANT:gAIL7 ant* line 3 stage 7 flowers and in ovule primordia but not in these tissues in L*er* flowers (Fig. 4f-i). *AIL7* mRNA did not accumulate as broadly in *ANT:gAIL7 ant* stage 7 flowers as *ANT* in wild type, because *ANT* mRNA was also present in stamen primordia (Elliott et al. 1996). *AIL7* mRNA was also not detected in the procambium of the inflorescence stem even though the *ANT* promoter is active here.

### Misexpression of ***AIL7*** using an ethanol inducible system results in some phenotypes similar to those present in ***ANT:gAIL7 ant*** line 10

**Fig. 5:**
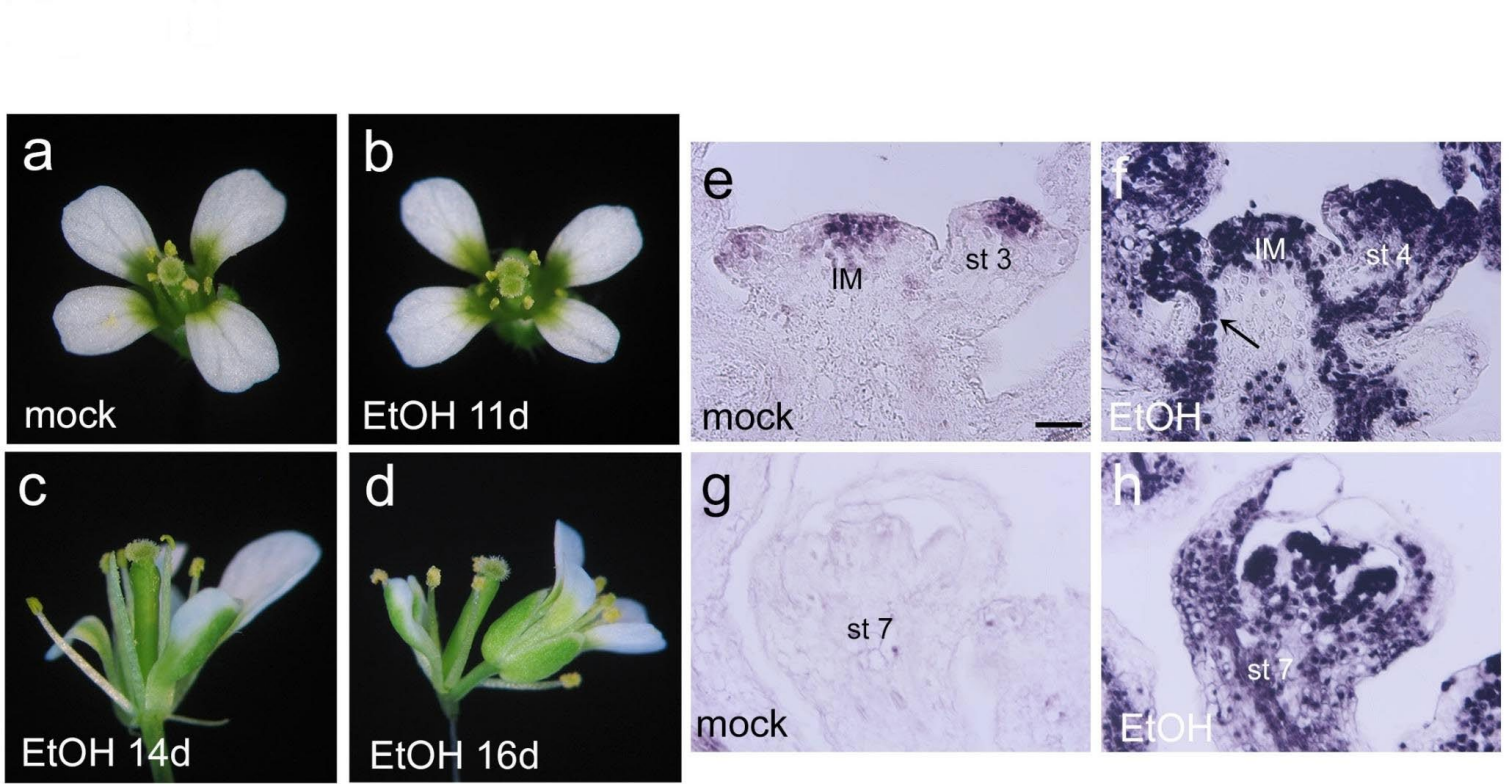
Ethanol treatment of *LFY:AlcR/AlcA:gAIL7-3’* leads to mosaic floral organs, alterations in floral organ number and secondary flowers **a**. Flower from a mock treated *LFY:AlcR/AlcA:gAIL7-3’* line 16 plant. **b-d**. Flowers from ethanol treated *LFY:AlcR/AlcA:gAIL7-3’* line 16 plants. **b**. Carpels with a split stigma were observed in a flower 11 days after ethanol treatment. **c**. Alterations in floral organ number and mosaic organs (petaloid sepal) were observed in a flower 14 days after ethanol treatment. **d**. A secondary flower was observed in this flower 16 days after ethanol treatment. **e-h**. In situ hybridization of *AIL7* mRNA in mock and ethanol treated *LFY:AlcR/AlcA:gAIL7-3’* line 16 plants. Arrow in f points to *AIL7* mRNA present in the procambium. Abbreviations: IM, inflorescence meristem; st 3, stage 3 flower; st 4, stage 4 flower; st 7, stage 7 flower. Size bar corresponds to 50 μm

To confirm that phenotypes seen in *ANT:gAIL7 ant* line 10 result from overexpression of *AIL7* in early stages of flower development, we created transgenic plants in which *AIL7* is expressed under the control of the *LEAFY* (*LFY*) promoter. *LFY* is expressed in flowers of stage 1–8 in a pattern somewhat similar to that of *ANT* (Weigel et al. 1992; Elliott et al. 1996). For these experiments, we used an ethanol inducible two component system in which the transcriptional activator *AlcR* is under control of the *LFY* promoter and a genomic copy of *AIL7* is under control of the *AlcA* promoter. The *AlcA* promoter is bound by AlcR only in the presence of ethanol. Of 16 *LFY:AlcR/AlcA:gAIL7* transgenic lines, six exhibited a strong phenotype, four exhibited a weak phenotype, and six exhibited no phenotype upon ethanol treatment. Mock treated inflorescences produced flowers with a wild-type appearance (Fig. 5a). Ethanol treatment of lines with a strong phenotype, as shown here for line 16, resulted in the production of stigmas with an oval or split appearance at 11 or 12 days after treatment, fused floral organs at 13–16 days after treatment, mosaic floral organs in whorls at 13–17 days after treatment, reduced numbers of floral organs at 15–16 days after treatment and double flowers at 16 days after treatment (Fig. 5b-d; Table 5). Mosaic organs were most commonly observed in whorls one and two (Table 5). First whorl sepals were often petaloid and occasionally stamenoid. Second whorl petals were often stamenoid and occasionally sepaloid. In addition, filaments were found in place of some floral organs in whorls one, two and three (Table 5). Several of these *AIL7* phenotypes resemble those found in *ANT:gAIL7 ant* line 10 including the production of mosaic floral organs, particularly petaloid sepals and the reduction in floral organ numbers.

**Table 5:** Floral organ counts of *LFY:AlcR/AlcA:gAIL7-3’* line 16

	EtOH-12d	EtOH-13d	EtOH-14d	EtOH-15d	EtOH-16d	EtOH-17d	H_2_O (12-17d)
**1st whorl**							
Se	4.00	3.50	2.40	2.00	1.57	4.00	4.00
Pe/Se; Se/Pe		0.67	1.60	0.71	1.14		
Fil		0.33	0.40	0.43	0.14		
Pe				0.14	0.14		
St/Se; Se/St					0.14	0.125	
Se-Pe/St					0.14		
St/Pe; Pe/St			0.20				
St-like				0.14			
Ca/Se					0.14		
**total**	**4.00**	**4.50**	**4.60**	**3.42**	**3.41**	**4.13**	**4.00**
**2nd whorl**							
Pe	4.00	3.50	2.80	2.14	1.28	3.88	4.00
Se/Pe						0.125	
St/Pe; Pe/St			0.20	0.29	0.14		
Fil			0.20	0.14			
**total**	**4.00**	**3.50**	**3.20**	**2.57**	**1.42**	**4.01**	**4.0**
**3rd whorl**							
St	5.40	5.83	5.80	5.29	5.00	5.50	5.69
St-like			0.20				
Fil			0.20	0.14	0.14		
**total**	**5.40**	**5.83**	**6.20**	**5.43**	**5.14**	**5.50**	**5.69**
**4th whorl**							
Ca	2.00	2.00	2.00	2.00	2.00	2.00	2.00
**total**	**2.00**	**2.00**	**2.00**	**2.00**	**2.00**	**2.00**	**2.00**
**Total all whorls**	**15.4**	**15.8**	**16.0**	**13.4**	**12.0**	**15.6**	**15.7**
% double flower	0%	0%	0%	0%	28.6%	0%	0%
% organ fusion	0%	50%	80%	0%	14%	0%	0%
% oval/split stigma	40%	0%	0%	0%	0%	0%	0%

We investigated *AIL7* mRNA expression in mock and ethanol treated *LFY:AlcR/AlcA:gAIL7* inflorescences by RT-qPCR and in situ hybridization. The strong *LFY:AlcR/AlcA:gAIL7* line 16 had *AIL7* mRNA levels that were 153 ± 42 fold higher in the ethanol treated as compared with the mock treated while the weak *LFY:AlcR/AlcA:gAIL7* line 5 had *AIL7* mRNA levels that were 30 ± 11 fold higher in the ethanol treated as compared with the mock treated. These results are consistent with *AIL7* overexpression being responsible for phenotypes present in *ANT:gAIL7 ant* line 10 flowers. Mock-treated *LFY:AlcR/AlcA:gAIL7* line 16 displayed a spatial pattern of *AIL7* expression matching that observed in wild type with expression in the center of stage 2 and 3 flowers and absent in stage 5–7 flowers (Fig. 5e, g) (Nole-Wilson et al. 2005). Ethanol-treated *LFY:AlcR/AlcA:gAIL7* inflorescences displayed a much stronger and broader pattern of *AIL7* mRNA accumulation than in the mock-treated inflorescences (Fig. 5f, h). *AIL7* mRNA was detected in both the sepal primordia and floral meristem of stage 4 flowers, persisted in older flowers of stage 5–7, and was present in the procambium of the inflorescence stem (Fig. 5f, h).

### AIL5 and AIL7 exhibit reduced transcriptional activation activities compared with ANT and AIL6 when bound to the consensus ANT DNA binding site

**Fig. 6:**
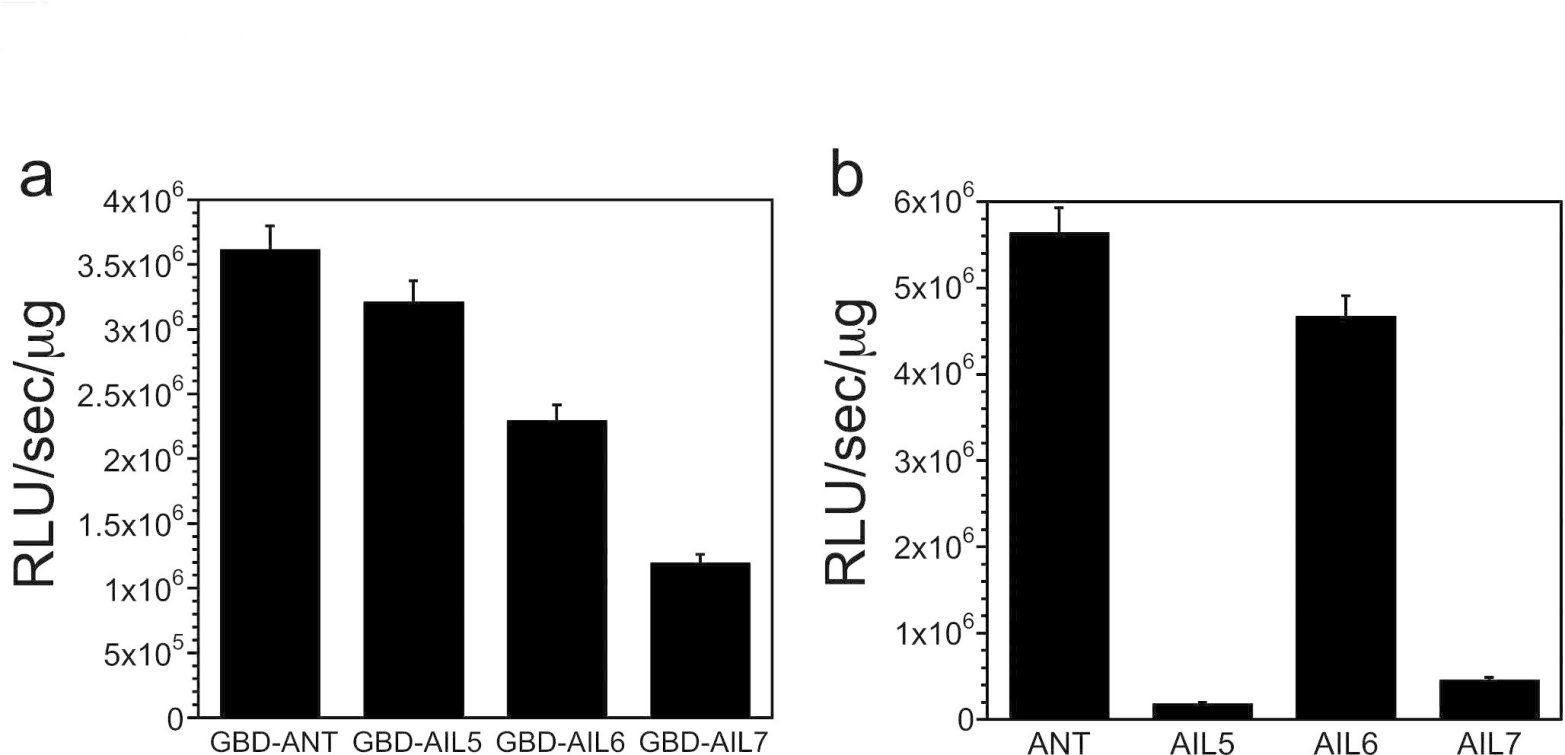
AIL5 and AIL7 exhibit reduced *lacZ* reporter gene activation in yeast when expressed under the control of the consensus ANT binding site (**a**) ANT, AIL5, AIL6, and AIL7 activate expression of the *lacZ* reporter gene to different degrees when expressed as fusions to the GAL4 DNA binding domain (GBD). The *lacZ* gene is under the control of three GAL4 binding sites and the TATA region of the *CYC1* promoter. (**b**) AIL5 and AIL7 exhibit much lower activation of the *lacZ* reporter gene than ANT and AIL6 when under the control of three ANT consensus binding sites and the TATA region of the *CYC1* promoter

Expression of *AIL5* and *AIL7* at levels similar to *ANT* levels in wild type results in a partial complementation of *ant-4*. The inability to fully complement *ant-4* suggests that the intrinsic gene regulatory activities of the AIL5 and AIL7 proteins are somewhat different than ANT. This could result from different DNA binding activities and/or differences in the abilities of these proteins to promote transcription. We investigated the transcriptional activation activities of ANT, AIL5, and AIL7 in yeast using a reporter line in which *lacZ* is under the control of three GAL4 binding sites and the TATA region of the *CYC1* promoter. ANT, AIL5, AIL6, and AIL7 were all able to activate transcription of *lacZ* when fused to the DNA binding domain of GAL4 (GBD), but the transcriptional activation activities of GBD-AIL6 and GBD-AIL7 were less than that of GBD-ANT and GBD-AIL5 (Fig. 6a). We also tested whether AIL5 and AIL7 could activate transcription when bound to the consensus ANT DNA binding site using a yeast reporter strain in which the *lacZ* reporter gene is under the control of a minimal *CYC1* promoter and three copies of the ANT consensus DNA binding site (Nole-Wilson and Krizek 2000; Krizek 2003). AIL5 and AIL7 displayed much lower levels of activation of the reporter gene as compared with ANT and AIL6 in this assay (Fig. 6b).

While a transcriptional activation domain has been mapped in ANT, this has not been investigated for AIL5, AIL6 or AIL7 (Krizek and Sulli 2006). To identify potential transcriptional activation domains in these AIL proteins, we used a neural network to predict the location and strength of acidic activation domains (Predictor of Activation Domains using Deep Learning in Eukaryotes, PADDLE) (Sanborn et al. 2021). Within ANT, PADDLE predicted an activation domain that is similar to that determined experimentally (amino acids 134–213) (Supplementary Fig. S3a). A significant acidic transcriptional activation domain and a strongly significant acidic transcriptional activation domain were predicted in the amino terminal regions of AIL6 and AIL7, respectively (Supplementary Fig. S3c, d). By contrast, no significant acidic activation domain was predicted for AIL5 (Supplementary Fig. S3b). Of all four proteins, PADDLE predicted that AIL7 has the strongest acidic activation domain with an activation Z-score above 7 (Supplementary Fig. S3d). The PADDLE transcriptional activation Z-scores for 53 amino acid tiled sequences are considered significant when greater than 4 and strongly significant when greater than 6.

## Discussion

### ***AIL5*** and ***AIL7*** expression levels similar to those of ***ANT*** in wild type inflorescences can partially complement ***ant***

Here we assess the ability of *AIL5* and *AIL7* to provide *ANT* function in flowers. Specifically, we examine floral organ number, petal size, anther locule number, and female sterility in *ant-4* compared with *ANT:gAIL5 ant-4* and *ANT:gAIL7 ant-4*. When expressed at levels similar to *ANT*, both *AIL5* and *AIL7* complement the anther locule defect. At these levels, we also observe that *AIL5* and to a greater extent *AIL7* partly rescue petal growth and that *AIL7* but not *AIL5* is able to produce seeds. Neither *AIL5* nor *AIL7* provide any rescue of floral organ number at this expression level. In contrast, *AIL6* largely complements *ant-4* when expressed at levels similar to *ANT* in wild type (Table 2) (Han and Krizek 2016). Thus, we find less rescue of *ant-4* by *AIL5* and *AIL7* compared with *AIL6*. The ability of *AIL5* and *AIL7* to rescue growth in some floral tissues but not others, suggests that different levels of ANT activity may be necessary in different developmental contexts. For example, promotion of anther growth leading to the development of four locules may require less ANT activity than floral meristem growth or petal growth.

### Protein activity differences likely contribute to ANT, AIL5 and AIL7 functional differences

Our results suggest that the functional differences between *ANT* and both *AIL5* and *AIL7* cannot be explained simply by expression differences alone. Thus, it is likely that differences in protein activity also contribute to functional differences. Differences in DNA-binding specificities or affinities, transcription activation activities, or protein-protein interactions could all potentially contribute to AIL proteins with different gene-regulatory capabilities.

The DNA binding specificities of ANT, AIL5, AIL6, and AIL7 have been determined previously using in vitro selection techniques. Two different SELEX (Systemic evolution of ligands by exponential enrichment) experiments have been performed with ANT and one with AIL5 (Supplementary Fig. S4a-c) (Nole-Wilson and Krizek 2000; Santuari et al. 2016). The AIL6 and AIL7 DNA binding site specificities were determined by DAP-Seq (Supplementary Fig. S4d, e) (O’Malley et al. 2016). The SELEX-determined DNA binding sites for ANT and AIL5 are similar, particularly for the ANT and AIL5 sites determined in Santuari et al. 2016 (Fig. S4 a-c). In addition, it has been shown that AIL5 can bind an ANT DNA binding site matching the other SELEX-identified ANT site shown in Supplementary Fig. S4a (Yano et al. 2009). Thus, the DNA binding specificities of ANT and AIL5 in vitro are similar. ChIP-Seq experiments on PLT2/AIL4, BBM/PLT4/AIL2, ANT, and AIL6 identified motifs similar to these in vitro determined DNA binding sites as overrepresented in the ChIP-Seq peaks, suggesting that such sequences are bound in vivo and that different AIL proteins exhibit similar DNA binding specificities in vivo (Horstman et al. 2015; Santuari et al. 2016; Krizek et al. 2020, 2021). However, the identified AIL7 DNA binding site is a shorter sequence than those determined for ANT, AIL5, or AIL6 (O’Malley et al. 2016). It retains three positions that are highly conserved in all of the binding sites (see positions 6, 13, 15 for the ANT site in Supplementary Fig. S4a). Whether AIL7 binds with strong affinity to the ANT consensus site remains to be determined.

Using reporter gene assays in yeast, we found lower transcriptional activation activities of AIL5 and AIL7 compared with ANT and AIL6, when bound to the ANT consensus binding site. AIL7 is predicted to contain a high activity transcriptional activation domain by PADDLE suggesting that the low level of transcriptional activation may result from weaker DNA binding to the ANT consensus binding site rather than an inherently weaker transcriptional activation activity. Although PADDLE did not predict an acidic transcriptional activity domain, the yeast data suggest that AIL5 has transcriptional activation activity.

The ability of *AIL5* to rescue all *ant* defects when expressed at higher mRNA levels than *ANT* suggests that reduced AIL5 activity in gene regulation can be compensated by higher levels of AIL5 protein. In contrast, floral organ number was not rescued in any *ANT:gAIL7 ant* lines, suggesting that even increased amounts of AIL7 protein may not result in the regulation of all ANT targets. Our work demonstrates that differences in both gene expression and protein activity confer the distinct functions of ANT compared with both AIL5 and AIL7. Further work is needed to measure the relative DNA-binding affinities of these proteins, characterize their transcription activation activities in planta, and identify protein-protein interaction partners that may contribute to the functional differences of these three proteins.

### Electronic supplementary material

Below is the link to the electronic supplementary material.

Supplementary Material 1

## Data Availability

The PADDLE output generated during the current study are available in a bitbucket repository, http://bitbucket.org/krizeklab.
