# Supplementary material for: Differences in both expression and protein activity contribute to the distinct functions of AINTEGUMENTA compared with AINTEGUMENTA-LIKE 5 and AINTEGUMENTA-LIKE 7
Source: Plant Mol Biol. 2023 Aug 22;113(1-3):75–88. doi: 10.1007/s11103-023-01374-0 (PMC10593615; doi:10.1007/s11103-023-01374-0)
Supplement: Supplementary file 1 — Supplementary Material 1 [file 11103_2023_1374_MOESM1_ESM.docx]

Supplementary Information

Differences in both expression and protein activity contribute to the distinct functions of AINTEGUMENTA compared with AINTEGUMENTA-LIKE 5 and AINTEGUMENTA-LIKE 7

Beth A. Krizek^1*^, Caitlin Boling Iorio^1^, Kevin Higgins^2^, and Han Han^1,3^

^1^Department of Biological Sciences, University of South Carolina, Columbia, SC 29208, USA

^2^Metagenetics LLC, Columbia, SC 29205, USA

^3^Current address: Co-Innovation Center for Sustainable Forestry in Southern China, College of Biology and the Environment, Nanjing Forestry University, Nanjing 210037, China

^*^Corresponding Author

Tel: 1-803-777-1876

Email: [krizek@sc.edu](mailto:krizek@sc.edu)


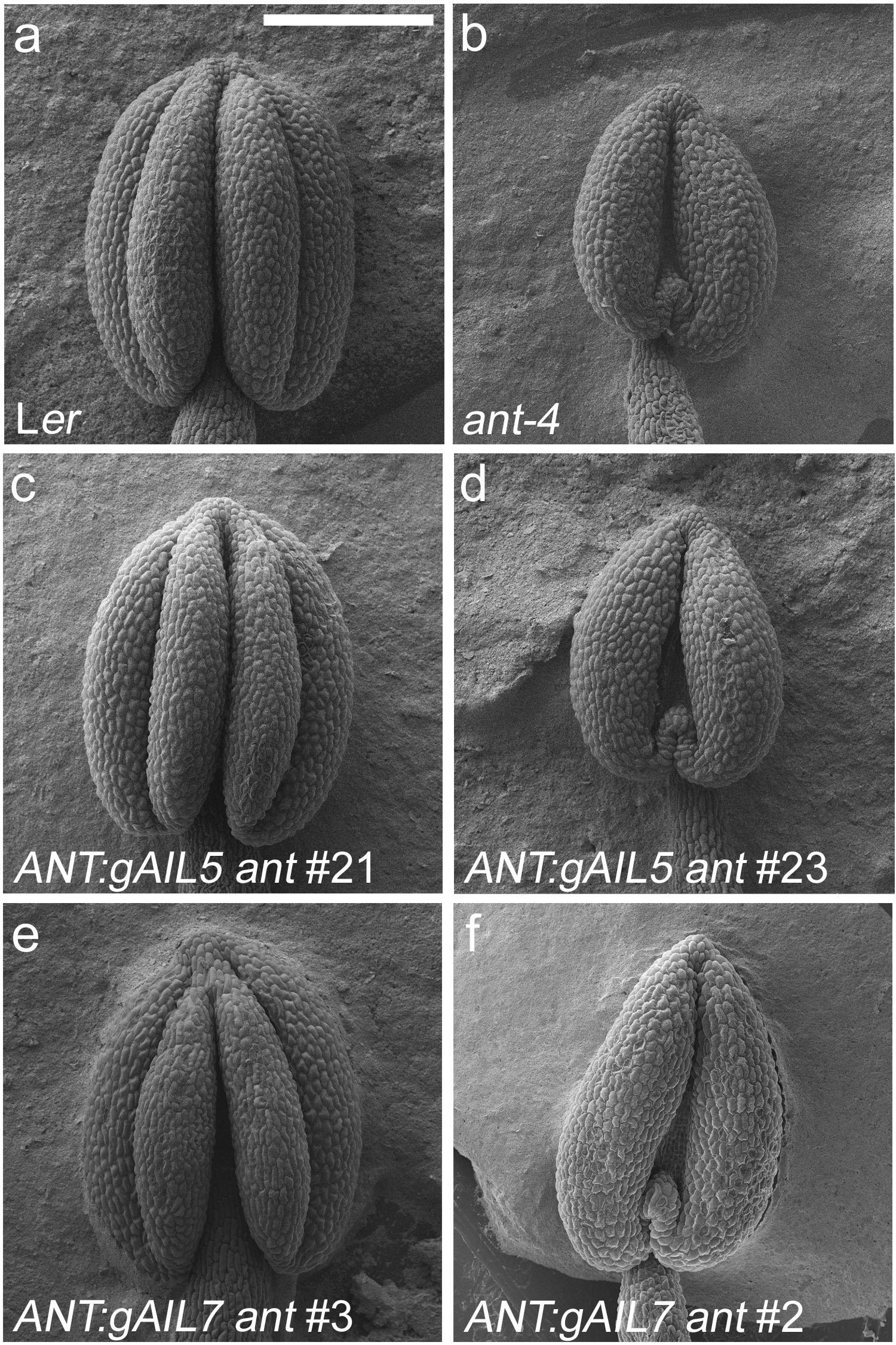


**Fig. S1** SEM micrographs of stamens from L*er* (a), *ant-4* (b), *ANT:gAIL5 ant-4* (c, d), and *ANT:gAIL7 ant-4* (e, f) flowers.

**
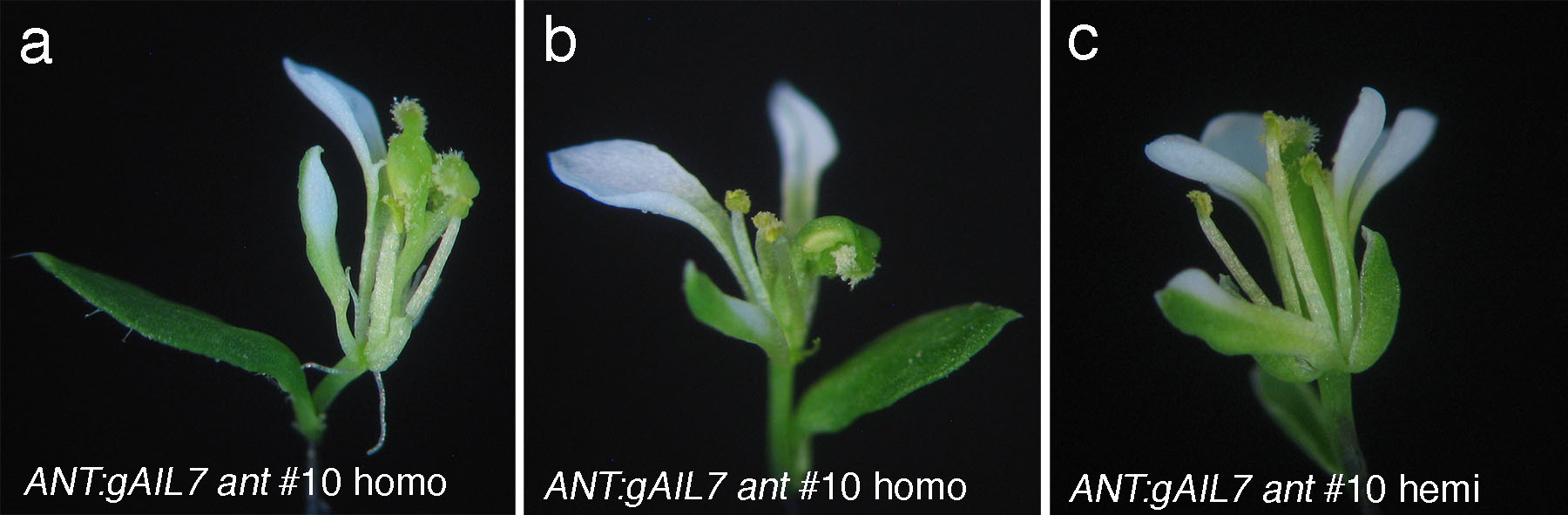
**

**Fig. S2** Flowers from *ANT:gAIL7 ant-4* line 10 homozygous (a, b) and hemizygous (c) plants.

**
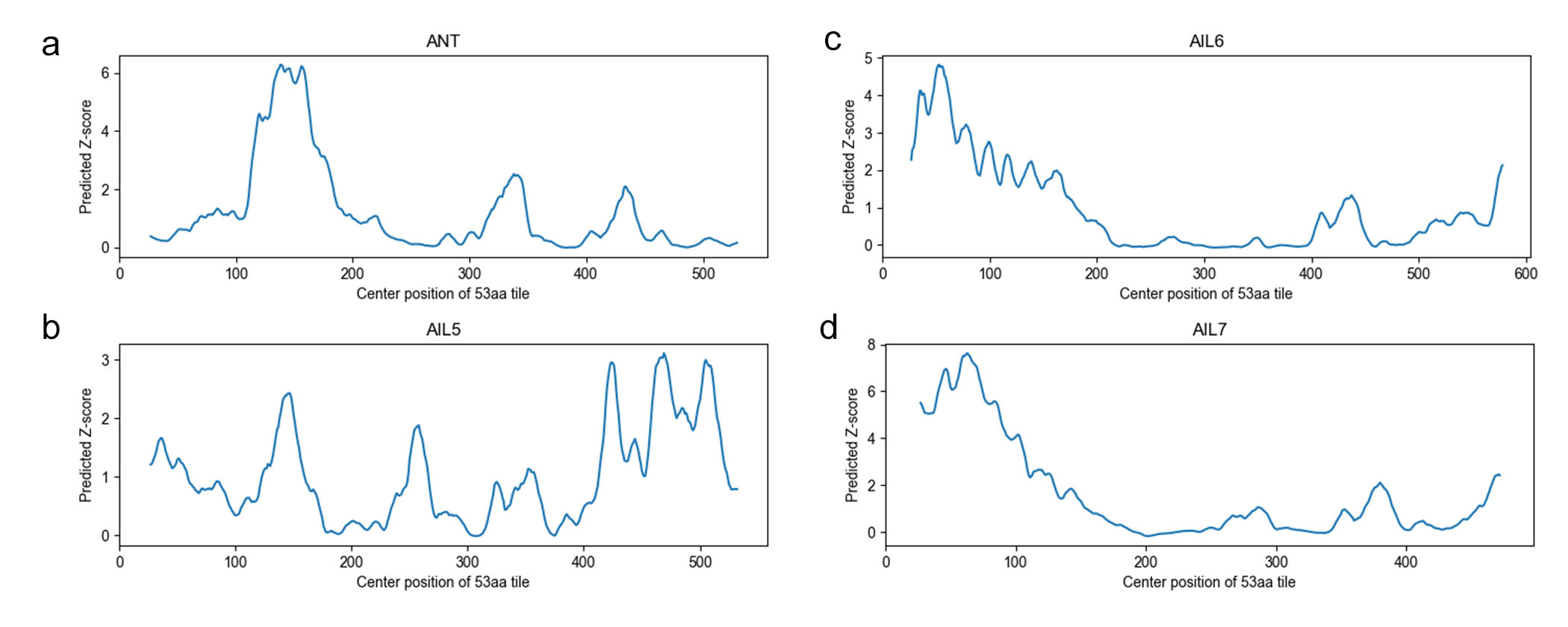
**

**Fig. S3** PADDLE predicted location and strength of acidic activation domains in ANT (a), AIL5 (b), AIL6 (c), and AIL7 (d). Activation Z-scores are considered significant when greater than 4 and strongly significant when greater than 6.


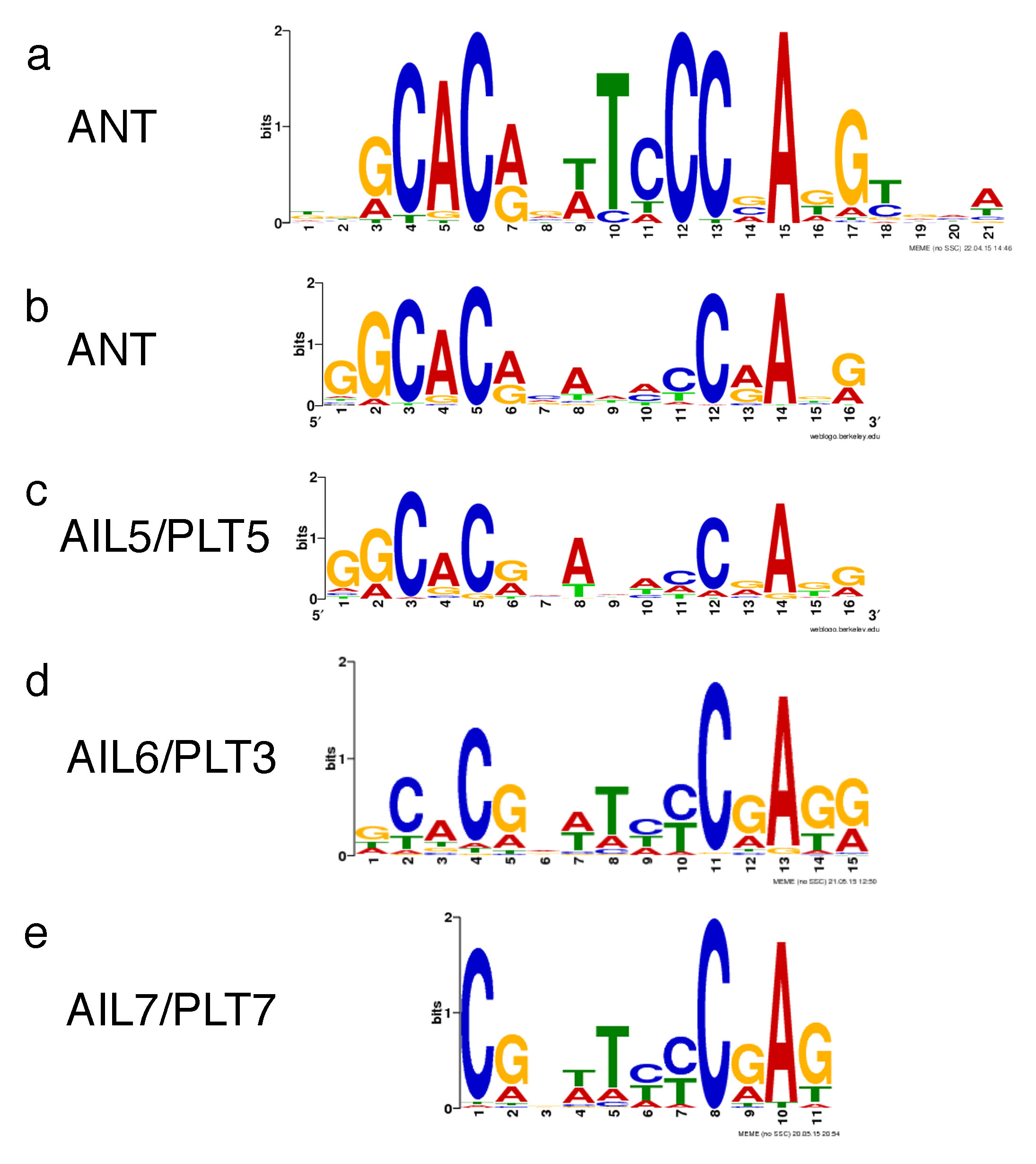


**Fig. S4** Sequence logos of the DNA binding specificities of ANT (a, b), AIL5 (c), AIL6 (d), and AIL7 (e) as determined by SELEX (a-c) and DAP-Seq (d,e) (Nole-Wilson and Krizek 2000; O’Malley et al. 2016; Santuari et al. 2016).
